# Supplementary material for: Chemokinergic and Dopaminergic Signalling Collaborates through the Heteromer Formed by CCR9 and Dopamine Receptor D5 Increasing the Migratory Speed of Effector CD4+ T-Cells to Infiltrate the Colonic Mucosa
Source: Int J Mol Sci. 2024 Sep 18;25(18):10022. doi: 10.3390/ijms251810022 (PMC11432204; doi:10.3390/ijms251810022)
Supplement: Supplementary file 1 [file ijms-25-10022-s001.zip › ijms-3179975-supplementary.pdf]

Supplementary material

Chemokinergic and dopaminergic signalling collaborates through the heteromer formed by CCR9 and dopamine receptor D5 increasing the migratory speed of effector CD4$^+$ T-cells to infiltrate the colonic mucosa

Javier Campos, Francisco Osorio-Barrios, Felipe Villanelo, Sebastián E. Gutierrez-Maldonado, Pablo Vargas, Tomás Pérez-Acle, Rodrigo Pacheco

Table S1. Peptides analogue to transmembrane segments of CCR9 and DRD5

Name	Sequence*
TM1D	VTAGLLTLLIVWTLLGNVLVSAAYGRKKRRQRRR
TM6D	RRRQRRKKRGYFKTLSVIMGVFVCCWLPFFILN
TM7D	VSETTFDIFVWFGWANSSLNPIIYGRKKRRQRRR
TM5C	KSAVLILKVTLGFFLPFMVMAFSYYGRKKRRQRRR
TM6C	RRRQRRKKRGYTITVLTVFIMSQFPYNSILVVQ
TM7C	ISFQVTQTIAFFHSSLNPVYGRKKRRQRRR
TM5C-FITC	^{FITC}-KSAVLILKVTLGFFLPFMVMAFSYYGRKKRRQRRR
TM6C-FITC	RRRQRRKKRGYTITVLTVFIMSQFPYNSILVVQ-^{FITC}
TM7C-FITC	^{FITC}-ISFQVTQTIAFFHSSLNPVYGRKKRRQRRR

*Transmembrane regions were predicted by 3D modeling of DRD5 (access code Q8BLD9.1) or CCR9 (access code Q9WUT7.1) following the criteria deduced from crystals of G-protein coupled receptors (William et al., 1992). To give a proper delivering of transmembrane peptides with the correct orientation in the plasma membrane, the TAT peptide (marked in red) was added in direct orientation (YGRKKRRQRRR) in the C-terminal of odd transmembrane segments and in the inverse orientation (RRRQRRKKRGY) in the N-terminal of even transmembrane segments. The TAT peptide is a cell-penetrating peptide derived from the transactivator of transcription protein of the human immunodeficiency virus. In addition, to avoid the formation of disulfide bridges, cysteines were replaced with serines (marked in green). FITC-coupled to some peptides is indicated in superscripts.

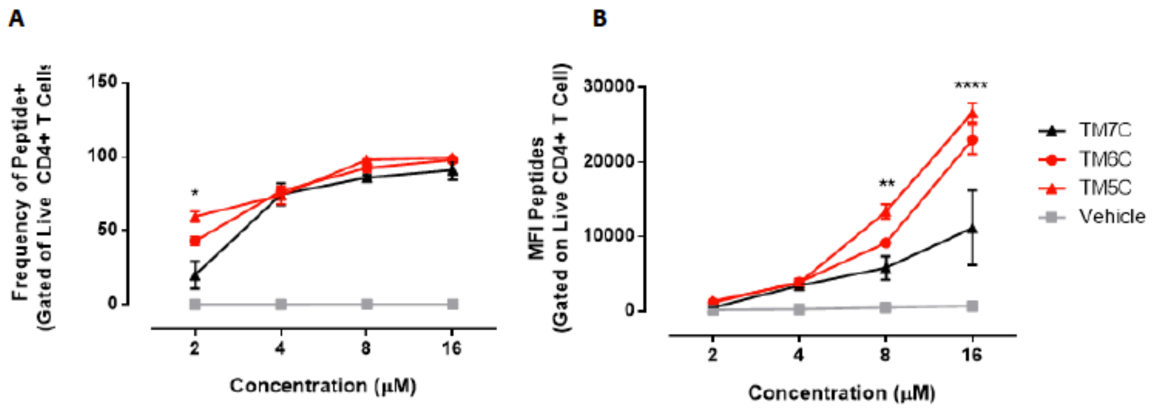

Figure S1. Analysis of binding of peptides analogues to TM segments from CCR9 to CD4$^+$ T-cells with gut tropism. Naïve CD4$^+$ T-cells were isolated from the spleen of wild-type mice (*Drd5*$^{+/+}$) and then activated with anti-CD3/anti-CD28 mAbs coated dynabeads in the presence of IL-2 and RA for 5 d to induce gut tropism. During the last 4 h, cells were treated with different concentraions of FITC-coupled peptides TM5C, TM6C or TM7C, or only DMSO as a control (vehicle). Afterwards, cells were stained for extracelular expression of CD4 and with a viability dye (ZAq), and peptide associated to the cells was analysed by flow cyometry. (**A**) Quantification of the percentage of cells displaying fluorescence associated to the indicated peptide in the CD4$^+$ live (ZAq$^-$) population. (**B**) Quantification of the density of peptide bound to the CD4$^+$ live (ZAq$^-$) population. Values are the mean fluorescence intensity (MFI) associated to the indicated peptide (FITC). (A, B) Data is represented as the mean ± SEM from three independent experiments. *, p<0.05; **, p<0.01; ****, p<0.0001 comparing TM5 or TM6 versus TM7 by one-way ANOVA followed by the Sidak's multiple comparisons post-hoc test.

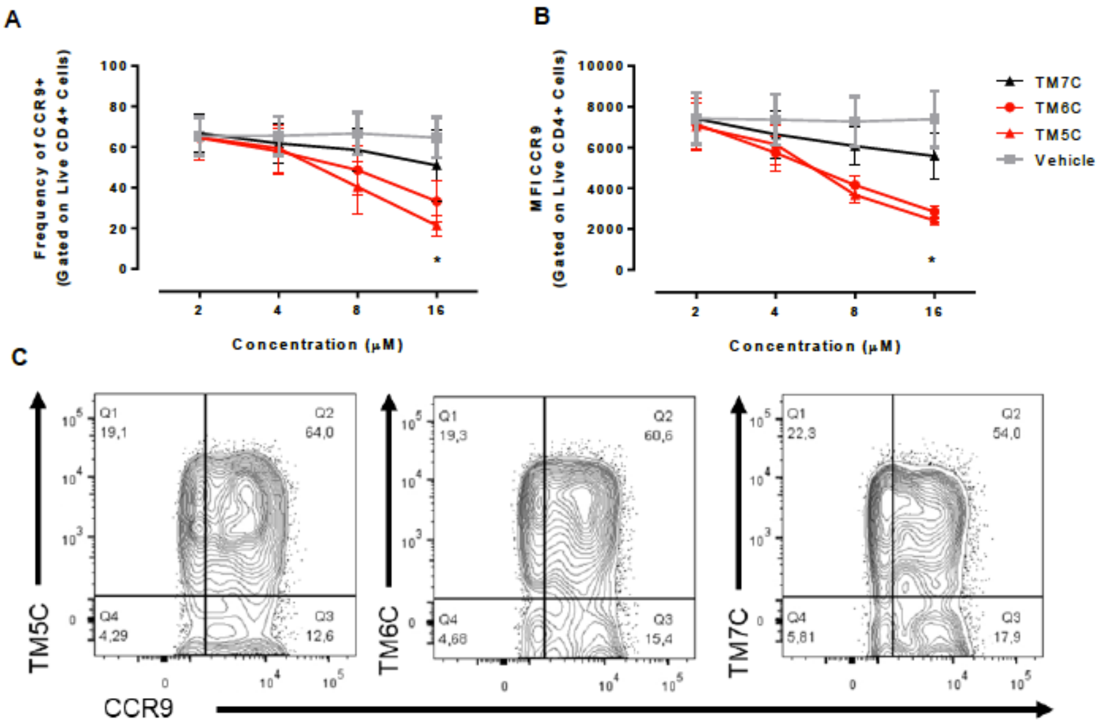

Figure S2. Peptides analogues to TM segments from CCR9 do not affect CCR9 expression on CD4^{+} T-cells with gut tropism. Naïve CD4^{+} T-cells were isolated from the spleen of wild-type mice (*Drd5*$^{+/+}$) and then activated with anti-CD3/anti-CD28 mAbs coated dynabeads in the presence of IL-2 and RA for 5 d to induce gut tropism. During the last 4 h, cells were treated with different concentraions of FITC-coupled peptides TM5C, TM6C or TM7C, or only DMSO as a control (vehicle). Afterwards, cells were stained for extracelular expression of CCR9, CD4 and with a viability dye (ZAq), and the CCR9 expression and peptide associated to live CD4+ T-cells was analysed by flow cyometry. (**A**) Quantification of the percentage of cells expressing CCR9 on the CD4^{+} live (ZAq^{-}) population. (**B**) Quantification of the density of CCR9 expression on the CD4^{+} live (ZAq^{-}) population. Values are the mean fluorescence intensity (MFI) associated to CCR9 immunostaining. (A, B) Data is represented as the mean ± SEM from three independent experiments. *, $p<0.05$ compared to the vehicle by one-way ANOVA followed by the Sidak's multiple comparisons post-hoc test. (**C**) Representative contour plots showing CCR9 expression and peptide (4 μM) binding. The percentages of cells in each quadrant are indicated.

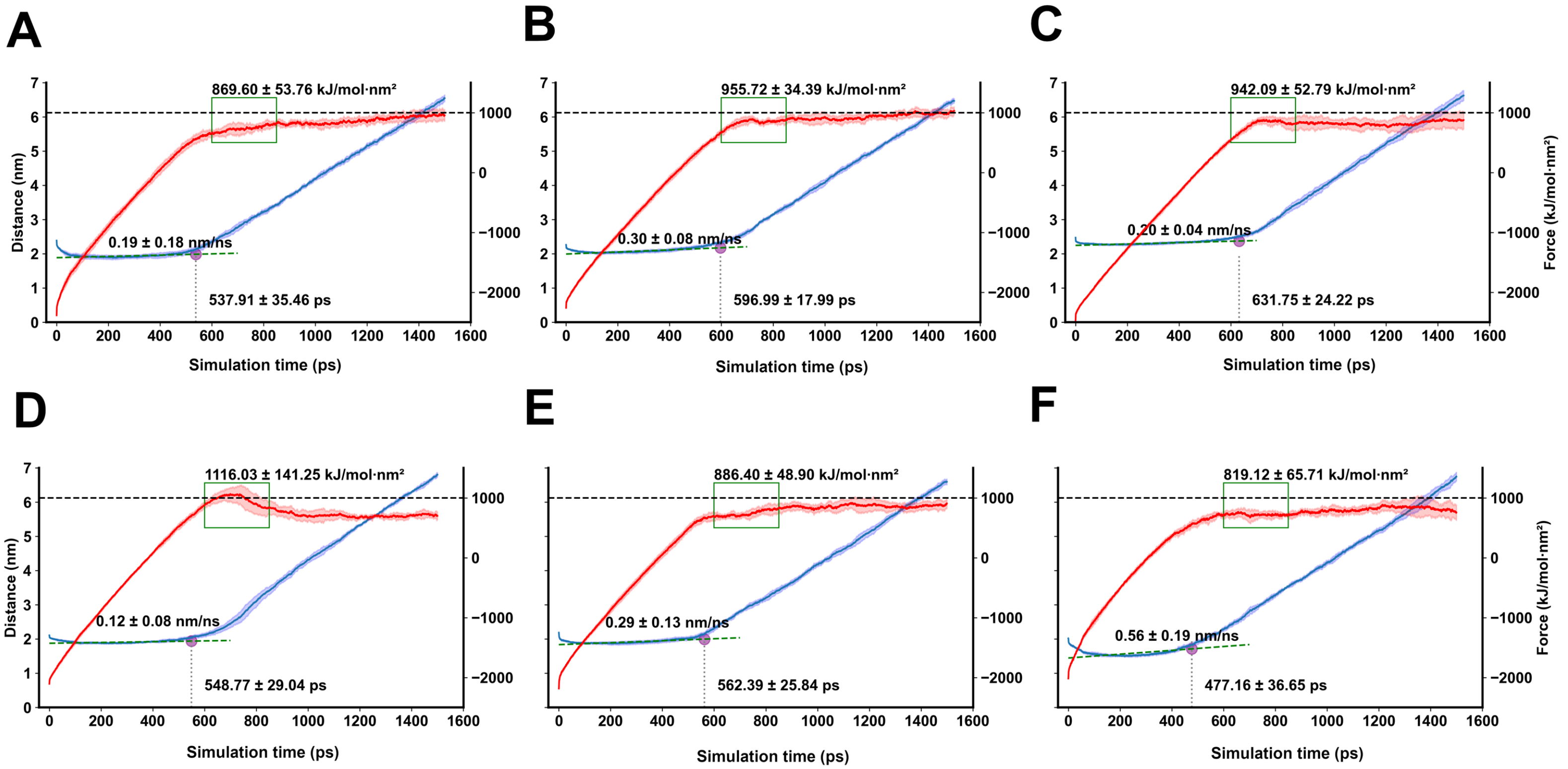

Figure S3. Analysis of the interaction of individual TM segments with the whole partner-protein by an umbrella pulling protocol. Plots of distance (blue lines, left y-axis) and force (red lines, right y-axis) versus simulation time. Solid line is the average from 6 independent simulations, and the shade represent standard deviation around average. The purple circle represents the transition point, when distance goes from nearly steady value to the change rate defined in the pull simulation (5 nm/ns). The green rectangle indicates the region of maximum force before the steady force at the value defined in the pull simulation (1,000 kJ/mol·nm²). (**A**) CCR9-TM1D; (**B**) CCR9-TM5D; (**C**) CCR9-TM6D; (**D**) DRD5-TM1C; (**E**) DRD5-TM5C; (**E**) DRD5-TM6C.
